# Separation of Native Allophycocyanin and R-Phycocyanin from Marine Red Macroalga *Polysiphonia urceolata* by the Polyacrylamide Gel Electrophoresis Performed in Novel Buffer Systems

**DOI:** 10.1371/journal.pone.0106369

**Published:** 2014-08-28

**Authors:** Yu Wang, Xueqin Gong, Shumei Wang, Lixue Chen, Li Sun

**Affiliations:** 1 College of Life Sciences, Yantai University, Yantai, Shandong, P. R. China; 2 College of Photo-electronic Information Science and Technology, Yantai University, Yantai, P. R. China; University of Hyderabad, India

## Abstract

Three buffer systems of Imidazole−Acetic acid, HEPES−Imidazole/Bis-tris and Bis-tris−HEPES−MES were designed based on the principle of discontinuous polyacrylamide gel electrophoresis (PAGE) for the native PAGE which could be performed in pH 7.0 and 6.5 in order to analyze and prepare the minor components of allophycocyanin (AP) and R-phycocyanin (R-PC) from marine red macroalga *Polysiphonia urceolata*. These AP and R-PC phycobiliproteins are easily denatured in alkaline environments. The obtained results demonstrated that the PAGE modes performed in the buffer systems of HEPES−Imidazole/Bis-tris and Bis-tris−HEPES−MES gave the satisfactory resolution and separation of AP and R-PC proteins. The absorption and fluorescence spectra of the AP and R-PC proteins which were prepared by the established PAGE modes proved that they maintained natural spectroscopic characteristics. The established PAGE modes may also provide useful references and selections for some other proteins that are sensitive to alkaline environments or are not effectively separated by the classical PAGE modes performed normally in alkaline buffer systems.

## Introduction

Phycobiliproteins (PBPs) are one type of photosynthetic pigment protein complexes found widely in cyanobacteria, red algae and some cryptophytes [1]–[4]. The PBPs from cyanobacteria and red algae are commonly divided into three types according to their light absorption properties: phycoerythrins (PE; λ_max_ = 490 to 570 nm), phycocyanins (PC; λ_max_ = 590 to 625 nm) and allophycocyanins (AP; λ_max_ = 645 to 655 nm) [3], [4]. In cyanobacteria and red algae, PBPs assemble in a supramolecular complex, named phycobilisome (PBS). The PBSs normally have two subdomains: peripheral rods and central cores. Both of the subdomains are made up of phycobiliproteins and their corresponding linker polypeptides (L) [2], [3]. PBSs attach on the external surface of thylakoids, where photosynthesis photoreaction takes place, and they function as the main photosynthetic accessories of the two organisms for sun light harvesting. Directional energy transfer in high efficiency from PBSs to photosystems depends absolutely on the structural intactness of ordered phycobiliprotein component organization of PBSs and the structural coupling of PBSs with the photosystems which are embedded in thylakoid membranes.

PBPs are oligomeric proteins and commonly composed of two kinds of chromophore-carrying subunits, α and β; the two subunits of α and β combine with each other to form monomers (αβ). Three monomers connect side by side to form a trimer, (αβ)_3_. In the assembly of PBS rod domains, two trimers of PE and PC further assemble face to face to build hexamers, (αβ)_3_L_R_(αβ)_3_, with the aid of rod linkers (L_R_), whereas AP commonly participates in PBS core construction in trimer form [2]–[4]. When PBSs are dissociated in diluting phosphate buffer solution [3], PCs and APs commonly exist in trimer, but PEs usually exist in hexamer. Hexameric PEs are stable much more than PCs and APs in diluting water solution since they carry linker polypeptides by which two trimeric PEs are connected [5], [6].

Light absorption and fluorescence emission of phycobiliproteins originate essentially from chromophores which are covalently linked by thioether bonds to cysteine residues at very specific sites, and the properties of the absorption and fluorescence spectra are determined by the type and number of the chromophores which a phycobiliprotein contains. C-phycoerythrins from cyanobacteria, such as C-PE I and C-PE II, B-phycoerythrin (B-PE) from unicellular red alga *Porphyridium cruentum* and R-phycoerythrin (R-PE) from some red macroalgae all contain two types of chromophores: red-colored phycoerythrobilin (PEB) and yellow-colored phycourobilin (PUB) [1]–[5]. This chromophore composition makes the PEs from cyanobacteria and red algae show absorption spectra with two or three peaks from 450 nm to 570 nm and fluorescent emission spectra with a maximum at ∼580 nm. C-phycocyanins (C-PCs) from cyanobacteria only contain one kind chromophore of blue-colored phycocyanobilin (PCB), whereas R-phycocyanins (R-PCs) from red algae also carry PEB as well as PCB [1]–[5]. Therefore, C-PCs commonly show absorption spectra with one peak at about 615–620 nm and R-PCs give absorption spectra with two peaks at ∼550 nm and ∼617 nm; nevertheless, both of the two kind PCs generally exhibit a strong fluorescence maximum at ∼640 nm. All of the allophycocyanins reported from cyanobacteria and red algae contain PCB [1]–[5]; they commonly show absorption maximum at ∼650 nm and a shoulder at ∼615 nm, and the fluorescence maxima of them lie generally in 660–680 nm. There have been no researches that report the APs of red algae deferent in spectral properties from those of cyanobacteria.

Besides the chromophores, light absorption and fluorescence emission magnitude of phycobiliproteins also depends quite on the arrangement of chromophores in phycobiliprotein trimers or hexamers and the microenvironments which the natural configuration of PBP complexes provide and maintain for their carrying chromophores [5]–[6]. Therefore, the factors that cause any changes of the natural configuration of multi-subunit PBP complexes, especially which related closely to the arrangement and configuration of the carried chromophores, may bring about the spectral variations of PBPs [6], [7]. In other words, any changes of PBP spectrum properties reveal the natural structure variations of PBP trimers and hexamers. Furthermore, for PBP trimers and hexamers the coefficients of light absorption and fluorescence emission will dramatically decrease when they are dissociated into monomers or subunits [5]–[6], [8]. Among the three type PBPs of PE, PC and AP, PEs generally exhibit higher stability than other two. This higher stability of PEs with respect to PCs and APs is commonly attributed to their linker-containing hexameric aggregates [3]–[6], (αβ)_3_L_R_(αβ)_3_ for the PEs from cyanobacteria and (αβ)_3_γ(αβ)_3_ for the PEs from red algae. For example, when C-PCs [9] and APs [10] are diluted to lower than 0.3 µM, the C-PC and AP trimers may dissociate to monomers and show dramatically decreasing in light absorption and fluorescence emission. In such a low concentration, these PBPs already exhibit their monomer spectra but not the trimer ones. Next, PBPs normally show their optimal spectra of light absorption and fluorescence emission in solution around neutral pH (usually within pH 6.5 to pH 7.5) [3], [3]–[6], but B-PE from *Porphyridium cruentum* [7], [11] and R-PEs from *Grateloupia turuturu* [7] and *Polysiphonia urceolata* [12] were proved to show the absorption and fluorescence spectra with negligible changes in pH from 4.5 to 9.5, and the spectral variations occurred beyond this pH scope were closely related to the structural changes of their hexameric aggregates [11]–[12]. These researches demonstrate that absorption and fluorescence spectra of PBPs can sensitively reveal structure and conformation variations of PBPs because the spectral properties of PBPs closely correlate to the high grade structures of PBP complexes and especially depend on those conformations supporting the natural micro-environments of chromophores [11]–[12]. Because of their excellent spectroscopic properties, such as high absorption coefficient and strong fluorescent emission, phycobiliproteins are widely used as fluorescent probes for immunodiagnostic protein and cell labeling in biological and medical tests [5], [6], [9]–[10], [13].

In the investigations and practical applications of PBPs, they are generally isolated and purified from alga materials by chromatography [14]–[15]. For example, B-phycoerythrin (B-PE) was prepared from *Porphyridium cruentum* by the DEAE-cellulose DE-52 chromatography which was combined with Sephadex G-100 gel filtration [16], and by Streamline-DEAE expanded-bed chromatography which was combined with DEAE-cellulose one [17]; the R-phycoerythrin (R-PE) from *Corallina elongata* was prepared by the hydroxyapatite chromatography which combined with Superdex 75 gel filtration [18], the R-PE of *Polysiphonia urceolata* was prepared by DEAE Sepharose chromatography [19] and also by expanded-bed phenyl-Sepharose Streamline chromatography which was combined with Q-Sepharose or hydroxyapatite chromatography [20]; the PBPs from cyanobacterium *Nostoc muscorum* were prepared by DEAE-cellulose DE-52 and hydroxyl apatite column chromatography [21], the PC from cyanobacterium *Aphanizomenon flos-aquae* was prepared by hydroxyapatite chromatography [22].

In addition, PBPs can be also prepared by electrophoresis [14]. For example, the PBPs of *Mastigocladus laminosus* were prepared by the PAGE with Tris−Boric acid buffer in pH 8.6 [23]. However when the PC and AP were prepared from cyanobacterium *Myxosarcina concinna* by the PAGE performed in the Tris−Boric acid buffer, the PBP bands showed their native color gradually fading during the PAGE. This indicated that the PC and AP proteins dissociated or denatured to a certain extent in the PAGE with a resolving gel in pH≥8.6 [24]. Instead of the Tris−Boric acid buffer, the PCs and APs of *Myxosarcina concinna* were acceptably prepared by employing the native PAGE performed in Tris−Barbital buffer systems in pH 7.5 [24]–[25]. Additionally the R-PE from *Palmaria palmata* could be prepared by the preparative PAGE with Tris−Gly buffer system in pH 8.8 [26], and similarly the PAGE performed in the Tris−Gly buffer system was used to analyze a R-PE component of the phycobilisomes from *Polysiphonia urceolata* [27]. These facts are consistent with the evidence that the PEs have relatively higher stability in alkaline conditions [7], [11]–[12]. But the R-PC component of the phycobilisomes from *Polysiphonia urceolata* had to be purified by the PAGE in the Tris−Barbital buffer system with a pH 7.5 resolving gel [28] from the R-PC fraction that still contained a trace number of R-PEs after its isolation by ion exchange chromatography. In other words, the R-PCs of the trace R-PE-containing fraction was prepared by the PAGE with the Tris−Barbital buffer system at the cost of some R-PCs losing because of their dissociating or denaturing throughout the PAGE. These examples of PBPs preparing by native PAGE demonstrate that the stability of PBPs during the native PAGE with the buffer systems in pH≥7.5 need to be given adequate consideration, even though native PAGE is indeed an efficient method fairly favorable to preparing especially some minor-content PBPs of algae in lab scale from the samples purified to a certain extent by chromatography.

Among classical discontinuous PAGE modes [29]–[31], the PAGE with Tris−Barbital buffer system is only one that is performed in a near neutral pH buffer system [32]. Even though in such a near neutral pH condition, the R-PC band still showed an observable decrease in its native color with the PAGE performing, implying the R-PCs at least had somewhat dissociation or denaturing during the PAGE. Therefore, the PAGE with Tris−Barbital buffer system is not a PAGE mode most favorable to the preparation of PBPs which are minor components in algae and also sensitive to alkaline environments. In this research, based on the principle of discontinuous PAGE [29]–[31], three buffer systems were elaborately designed for the native PAGE which could be performed in pH 7.0 and 6.5. The PAGE carried out in these buffer systems was tested by the analysis and preparation of R-PC and AP fractions purified partially by chromatography from *Polysiphonia urceolata*. By investigations on the composition of the individual buffer systems and on the combination of the gel buffers with electrode ones, desired effects of the PAGE in the two buffer systems on the separation of the minor R-PC and AP were achieved in cooperation with regulation of the gel concentrations.

## Materials and Methods

### Phycobiliprotein samples

All phycobiliproteins employed as the samples of native PAGE were prepared from a marine red macroalga *P. urceolata* which is widespread on the local seaside around Yantai city in the area of Northern Yellow Sea of China. The alga specimens were collected at the beach within the district under the jurisdiction of the city government. For the marine algae which are not protected organism species and are used as samples only for teaching and academic or scientific investigations, there are no regulations on the specimen collection limitation of them from the city government and other organizations of biological diversity protection. Therefore, the specimen collection of *P. urceolata* used in the research is not required to apply for a specific permission to government departments or related organizations. In addition, there are no other protected organism species included in this investigation.

R-PE was purified by the ion exchange chromatography on DEAE-Sepharose from the R-PE fraction which was isolated by the gel filtration on Sephadex G-150 from the phycobiliprotein extract of *P. urceolata* [33].

R-PC and AP samples were obtained by the ion exchange chromatography on DEAE-Sepharose from the R-PC fraction of gel filtration [33]–[34]. The AP component of the samples was adequately enriched because most of the R-PC component was isolated by the DEAE-Sepharose chromatography.

### Buffer systems of native gel electrophoresis

In order to optimize the analysis and preparation of the minor phycobiliprotein components of R-PC and AP from *P. urceolata* by native PAGE, different buffer systems of resolving gels in pH 7.0 and pH 6.5 and stacking gels in pH 6.0 and pH 5.4 were designed based on the principle of discontinuous PAGE. The buffer systems were tested and optimized in cooperation with the regulation of resolving gel concentration (T%) in a range from 5.5% to 10.5% and of stacking gel concentration in 3% and 4%; the cross-linking degree (C%) of resolving and stacking gels were 3% and 20%, respectively. After the electrophoresis, the gel bands were visualized first in native color pattern or in grey scale pattern with and without red filter, then in fluorescent pattern under UV-light at 365 nm and finally in stained pattern of Coomassie Blue G-250. When the red filter is used, red R-PE bands are filtrated and then the bands of AP in sky blue and R-PC in blue are distinguished easily on the photograph with respect to that without the red filter. The native PAGE with This−Gly and Tris−Barbital buffer systems [29]–[32] were used as controls in the experiments.

The buffer systems employed in the native PAGE were as following:

1. This−Gly buffer system: Tris−HCl buffers of the resolving gel in pH 8.8 contained Tris 0.375 M, HCl 70 mM and the stacking gel in pH 6.8 contained Tris 63 mM, HCl 59 mM; the Tris−Gly electrode buffer in pH 8.3 was composed of Tris 4.9 mM and Gyl 38.4 mM.
2. Tris−Barbital buffer system: the resolving gel buffer of Tris-HCl in pH 7.5 contained 93 mM Tris and 76 mM HCl; the stacking gel buffer of Tris−HCi in pH 5.5 contained 63 mM Tris and 62 mM HCl; the Tris−Barbital electrode buffer consisted of 6.9 mM Tris and 37 mM Barbital.
3. Imidazole−Acetic acid buffer systems: resolving gel buffers in pH 7.0/pH 6.5 contained imidazole 0.40 M and acetic acid 0.217/0.309 M; stacking gel buffers in pH 6.0/pH 5.4 contained imidazole 0.16 M and acetic acid 0.155/0.188 M. Tricine−Imidazole electrode buffers were: Imidazole−Acetic acid buffers in pH 6.5/pH 6.3 (imidazole 40 mM and acetic acid 31/35 mM) were used as anode buffers, and Tricine−Imidazole buffers in pH 6.5/pH 6.3 (Tricine 40 mM and imidazole 1.54/87 mM) were used as cathode buffers.
4. HEPES−Imidazole/Bis-tris buffer systems: resolving gel buffers of HEPES−Imidazole in pH 7.0/pH 6.5 consisted of HEPES 0.30 M and imidazole 122/32 mM, and those of HEPES−Bis-tris in pH 7.0/pH 6.5 consisted of HEPES 0.30 M and Bis-tris 0.32 M/54.7 mM; stacking gel buffers of HEPES−Imidazole in pH 6.0/pH 5.4 contained HEPES 0.20 M and imidazole 5.95/1.44 mM, and those of HEPES−Bis-tris in pH 6.0/pH 5.4 contained HEPES 0.20 M and Bis-tris 7.62/1.54 mM. Besides the Tricine−Imidazole electrode buffers, Bis-tris−Acetic acid buffers in pH 6.5/pH 6.3 (Bis-tris 40 mM and acetic acid 18/23 mM) and Tircine−Bis-tris buffers in pH 6.5/pH 6.3 (Tricine 40 mM and Bis-tris 2.61/1.32 mM) were respectively used as anode and cathode buffers (Tircine−Bis-tris electrode buffers).
5. Bis-tris−HEPES−MES buffer systems: resolving gel buffers of Bis-tris−HEPES−MES in pH 7.0/pH 6.5 consisted of Bis-tris 0.375 M, HEPES 0.263/0.187 M and MES 0.022/0.214 M; a stacking gel buffer in pH 6.0 consisted of Bis-tris 0.20 M, HEPES 0.20 M and acetic acid 0.145 M, and that in pH 5.4 contained Bis-tris 0.20 M, MES 50 mM and acetic acid 0.204 M. The Tircine−Bis-tris buffers in pH 6.5 and pH 6.3 were employed as electrode solutions.

### Spectrum measurement

Absorption spectra of prepared phycobiliprotein samples from corresponding steps were recorded in pH 7.0 phosphate buffers with UV-1900 spectrometer (Purkinje General Instrument Co. Ltd., Beijing, China) [28]. Fluorescence emission spectra of the purified R-PC and AP prepared by the native PAGE were measured by LS-55 fluorescence spectrophotometer (PerkinElmer, Inc., San Jose, California, USA) in pH 7.0 phosphate buffers.

## Results

**Figure 1:**
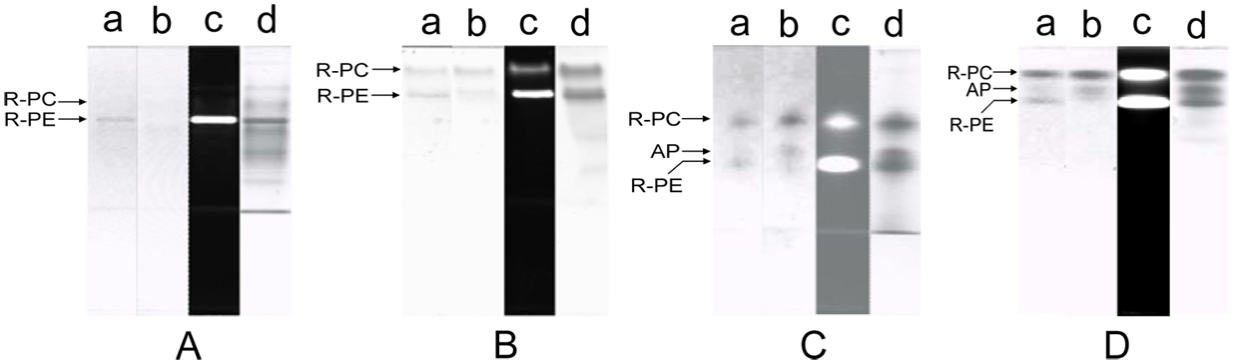
The native PAGE of the R-PC and AP sample performed in Tris−Gly (A), Tris−Barbital (B), Imidazole−Acetic acid (C) and Bis-tris−HEPES−MES (D) buffer systems. (A) the Tris−Gly PAGE with a resolving gel of 6.5% in pH 8.8 Tris−HCl buffer, a staking gel of 3% in pH 6.8 Tris−HCl buffer and an electrode buffer of Tris−Gly in pH 8.3; (B) the Tris−Barbital PAGE with a resolving gel of 6.5 in pH 7.5 Tris−HCl buffer, a staking gel of 3% in pH 5.5 Tris−HCl buffer and an electrode buffer of Tris−Barbital in pH 7.0; (C) the Imidazole−Acetic acid PAGE with a resolving gel of 6.5% and a staking gel of 3.0% in pH 6.5 and 5.4 Imidazole−Acetic acid buffers and an electrode buffer of Tricine−Imidazole in pH 6.3; (D) the Bis-tris−HEPES−MES PAGE with a resolving gel of 7.0% in pH 6.5 Bis-tris−HEPES−MES buffer, a stacking gel of 4.0% in pH 6.0 Bis-tris−HEPES buffer and electrode buffers of Bis-tris−Tricine and Bis-tris−Tris in pH 6.3. Protein band patterns were visualized in grey scale without (a) and with (b) red filter, in fluorescence (c) under UV-light at 360 nm and by Coomassie Blue G-250 staining (d). The used sample was the R-PC and AP fraction from the ion exchange chromatography, and the same amount of the sample was equally loaded on each mode of the PAGE.

The native PAGE of the R-PC and AP fraction from the ion exchange chromatography was performed in Tris−Gly, Tris−Barbital, Imidazole−Acetic acid and Bis-tris−HEPES−MES buffer systems, and the results were showed in Fig. 1. In the experiments, when the Tris−Gly PAGE was carried out for ∼45 min the R-PC and AP bands were found to begin their color decreasing, and the color bands of them almost completely faded after the electrophoresis ran for ∼60 min. Consequently, no fluorescent bands of the R-PC and AP were visualized in Fig. 1A (lane c) except for that of the R-PE. The bands of the native R-PC and AP trimers were also not clearly observed in grey scale without (Fig. 1A lane a) and with red filter (Fig. 1A lane a) except for the band of the R-PE hexamers (Fig. 1A lane a). These facts indicated that the Tris−Gly PAGE in pH≥8.8 [30] made the R-PC and AP have structural destroys to such a serious degree that the absorption and fluorescence of the R-PC and AP trimers were virtually lost in the electrophoresis performing. Among the bands (Fig. 1A lane d) showed by Coomassie Blue G-250 staining, however, there were ones which corresponded to the remnants of the R-PC and AP complexes created in the electrophoresis.

Likewise, the R-PC and AP bands also exhibited color fading to some extent with the Tris−Barbital PAGE running. Fortunately, as shown in Fig. 1B, the R-PC was still be observed as a thin fluorescent band other than the R-PE (lane c); moreover the R-PC band in native state was also observed in grey scale band patterns without (lane a) and with red filter (lane b), but the AP band could not be resolved. Nevertheless, the color fading and the fluorescence decreasing definitely indicated that the configuration of the R-PC and AP trimers, especially which fairly related to the optimal microenvironments of chromophores, was affected to a quite extent so that the fluorescence emission from their trimers adequately decreased with the Tris−Barbital PAGE performing in pH 7.5 to 8.0 [30]. Compared with the band pattern of the Tris−Gly PAGE (Fig. 1A lane d), the R-PC and R-PE bands of the Tris−Barbital PAGE (Fig. 1B lane d) were thicker and the bands before the R-PE were relatively fewer. This was seemly corresponding to the degree of the color and fluorescence decreases showed in the PAGE of Tris−Gly and Tris−Barbital buffer systems. The absorption and fluorescence decrease of the R-PC from *P. urceolata* during the PAGE in pH≥8.0 is consistent with the experimental results of the relationship between pH environments and the spectral properties of the R-PC (S.1 and S.2).

In contrast with the two PAGE methods mentioned above, the R-PC and AP exhibited no obvious fading in color during the native PAGE performed in the Imidazole−Acetic acid buffer system with a resolving gel in pH 6.5, a stacking gel in pH 5.4 and an electrode buffers in pH 6.3. As a result, the bands of the R-PC and AP trimers were observed definitely in grey scale band patterns (Fig. 1C, lane a and b), especially on the lane (lane b) where the R-PC and AP bands were strengthened by filtrating the R-PE one with a red filter. The band patterns in fluorescence (lane c) and Coomassie Blue G-250 staining (lane d) were essentially consistent with those in grey scale (lane a and b) except that the fluorescent band of the AP trimer was not imaged satisfactorily because the R-PE band with much stronger yellow fluorescence virtually interfered with the adjacent AP band with weaker red fluorescence. When the resolving gel of 8%–16% in pH 7.0 was employed, the AP band occurred before the R-PE one (S. 3). In addition, as shown in Fig. 1D, the PAGE with the Bis-tris−HEPES−MES buffer system seemed more favorable to preventing the R-PC and AP from color fading and fluorescence decreasing as well as to them separating (see following sections in detail).

**Figure 2:**
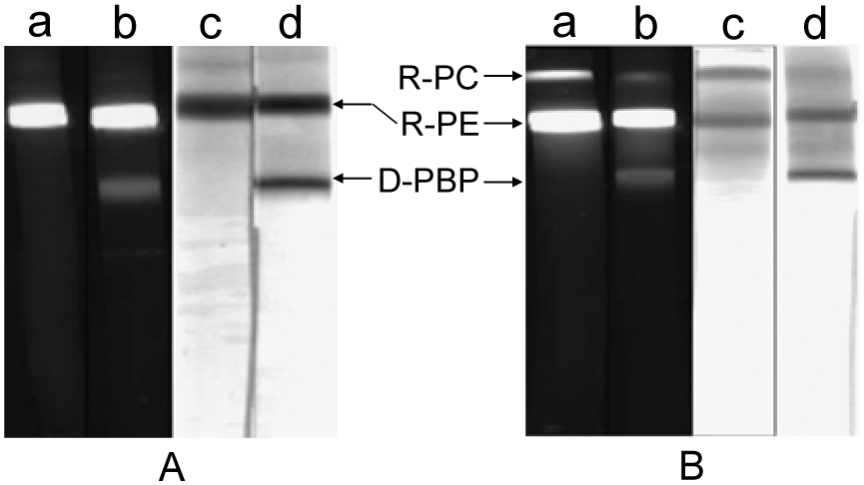
The native PAGE of the purified R-PE (A) and the R-PC sample (B) performed in HEPES−Imidazole and Imidazole−Acetic acid buffer systems. The PAGE with the resolving gel of 9% in pH 6.5 and the stacking gel of 4% in pH 5.4 was performed respectively in: (a) and (c) HEPES−Imidazole buffers; (b) and (d) Imidazole−Acetic acid buffers. Imidazole−Acetic acid and Tricine−Imidazole buffers in pH 6.3 were employed respectively as anode and cathode solutions. The protein bands were visualized under UV-light (a and b) at 360 nm and by Coomassie Blue G-250 staining (c and d). D-PBP represented the dissociated phycobiliprotein. The R-PC sample used in the PAGE was the R-PC fraction obtained by the ion exchange chromatography.

Although the PAGE in the Imidazole−Acetic acid buffer system seemed to give a satisfactory component separation of the R-PC and AP sample, by contrast with the native PAGE performed in HEPES−Imidazole buffer systems, as showed in Fig. 2, the R-PC sample (Fig. 2B) and even the purified R-PE (Fig. 2A) both obviously exhibited a protein band in the front part of the PAGE carried out in the Imidazole−Acetic acid buffer system (Fig. 2 lane b and d). This protein band was assumed to originate from the R-PE hexamer and R-PC trimer dissociating to monomers or subunits, named dissociated phycobiliprotein (D-PBP), for the fluorescent intensity of the protein band severely decreased. The R-PC and R-PE dissociation is most possibly brought about by the higher imidazole concentration of the Imidazole−Acetic acid buffer systems because the same electrode buffer is employed in both types of the native PAGE. These results indicated that the HEPES−Imidazole buffer system was more powerful to prevent phycobiliproteins from the dissociation in processes of their analysis and preparation by the two native PAGE modes. Nevertheless, the protein bands showed in the PGAE of the HEPES−Imidazole buffer system are a little broader than those in that of the Imidazole−Acetic acid buffer system (the result not shown), especially with the PAGE time prolonging; this inevitably decreases the band resolution and separation of the PAGE carried out in the HEPES−Imidazole buffer system.

**Figure 3:**
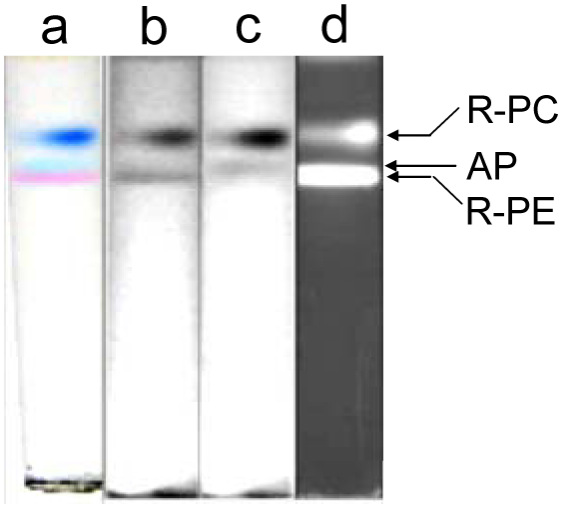
The native PAGE of the R-PC and AP sample performed in the HEPES−Imidazole buffer. The PAGE had a resolving gel of 7% in pH 6.5 HEPES−Imidazole buffer and a stacking gel of 4% in pH 5.4 Bis-tris−Acetic acid buffer; the Tricine−Bis-tris electrode buffers in pH 6.3 were employed as anode and cathode solutions. (a) the bands in natural colors, (b) the bands of native state in grey scale without red filter, (c) the bands of native state in grey scale with red filter and (d) the bands in fluorescence under UV-light at 365 nm. The R-PC and AP sample used in the PAGE was the R-PC fraction from the ion exchange chromatography.

In the research, to improve the native PAGE a Bis-tris−Acetic acid buffer system was designed for replacing the Imidazole−Acetic acid buffers. Unfortunately when the PAGE in the Bis-tris−Acetic acid buffers was carried out for about 90 min, it showed the current down to 2–3 mA and the voltage up to the highest scale so that the protein bands almost stop running. This defect limited the time lasting of the PAGE which could be performed in the state with adjustable electric current or voltage. Therefore, the PAGE performed in the Bis-tris−Acetic acid buffers always gave results where the protein bands were insufficiently resolved (the result not shown) though the loaded sample was stacked so efficiently that all of the various proteins in different color ran into the resolving gel as an indistinguishable narrow band. As showed in Fig. 3 where the three bands of the R-PC sample from the gel filtration were acceptably resolved, however, the disadvantage of the PAGE showed in the Bis-tris−Acetic acid buffer systems was effectively overcome by substituting resolving gel buffers of the Bis-tris−Acetic acid systems for those of the HEPES−Imidazole systems.

**Figure 4:**
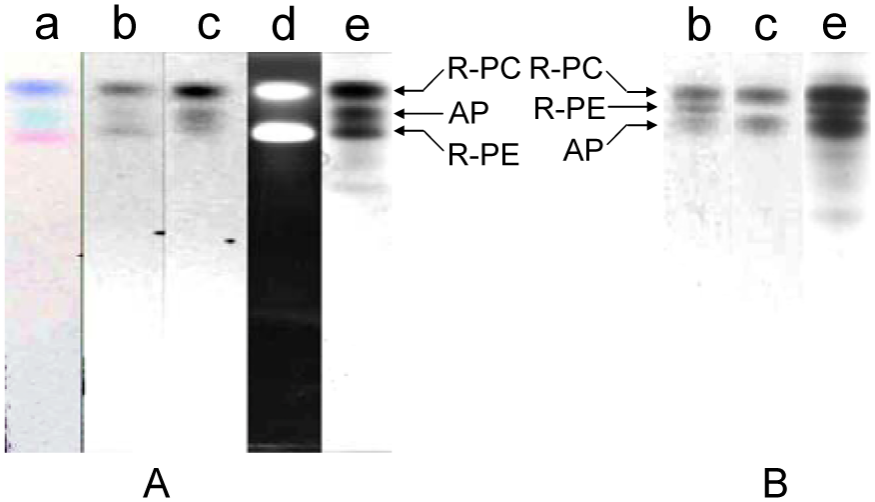
The resolution of R-PC and AP in Bis-tris-HEPES-MES buffer system with the stacking gel. The PAGE with the stacking gel of 4% in pH 6.0 Bis-tris−HEPES−Acetic acid buffer was performed respectively with: the resolving gel of 7% (A) and of 9.5% (B) in pH 7.0 (A) and pH 6.5 (B) Bis-tris−HEPES−MES buffers; the Tricine−Bis-tris electrode buffers in pH 6.5 (A) and pH 6.3 (B) were used in the PAGE. (a) the bands in natural colors, (b) the bands of native state in grey scale without red filter, (c) the bands of native state with red filter, (d) the bands in fluorescence under UV-light at 365 nm and (e) the bands stained by Coomassie Blue G-250. The sample used in the PAGE was the R-PC and AP fraction obtained by the ion exchange chromatography.

**Figure 5:**
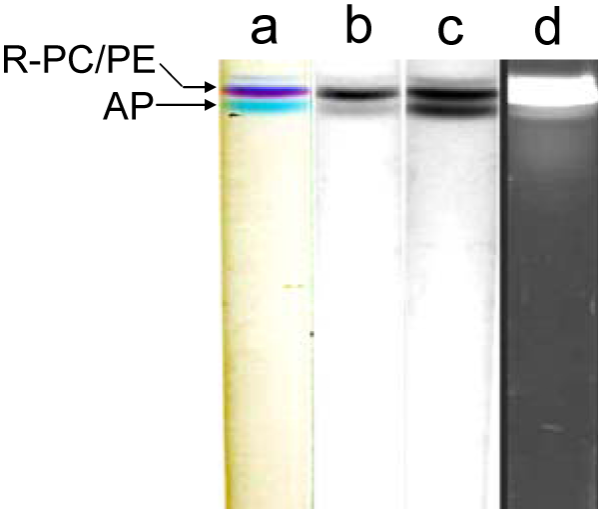
The resolution of AP in Bis-tris-HEPES-MES buffer system without stacking gel. The PAGE was carried out with a resolving gel of 9% in pH 6.5 Bis-tris−HEPES−MES buffer and with no stacking gel exclusively for the AP preparation. (a) the bands in natural colors, (b) the bands of native state in grey scale without red filter, (c) the bands of native state in grey scale with red filter, (d) the bands in fluorescence under UV-light at 365 nm and (e) the bands stained by Coomassie Blue G-250. The Tricine−Bis-tris electrode buffers in pH 6.3 were used in the PAGE. The sample used in the PAGE was the R-PC and AP fraction from the ion exchange chromatography.

In order to further improve the separation effect of the native PAGE used for the preparation of R-PC and AP components, Bis-tris−HEPES−MES buffer systems were designed and tested by analysis of the AP fraction from the ion exchange chromatography. As showed in Fig. 4, the three major components of R-PC, AP and R-PE were distinctly separated by the native PAGE performed in the Bis-tris−HEPES−MES systems with two resolving gels different in concentration. In the 7% resolving gel the AP band of the AP sample occurred between the R-PC and the R-PE. This order was the same as that when the proteins ran from the stacking gel into the resolving gel. At the beginning of the proteins running in the 9.5% gel, the AP band also moved in front of the R-PC and behind the R-PE, but with the PAGE performing the AP band gradually ran through the R-PE and then migrated in front of the R-PE band which was situated as the narrowest band before the R-PC and after the AP. Furthermore, when the three phycobiliproteins were separated by the PAGE with a 9% resolving gel but without a stacking gel, as showed in Fig. 5, the AP protein was almost the first bend running into the gel from the loaded sample. Consequently, it was rapidly resolved and clearly separated from the R-PC and R-PE both of which were located behind as an overplayed band. This may let the exclusive preparation of the AP component by the PAGE a little more straightforward for some special purposes of AP investigations.

**Figure 6:**
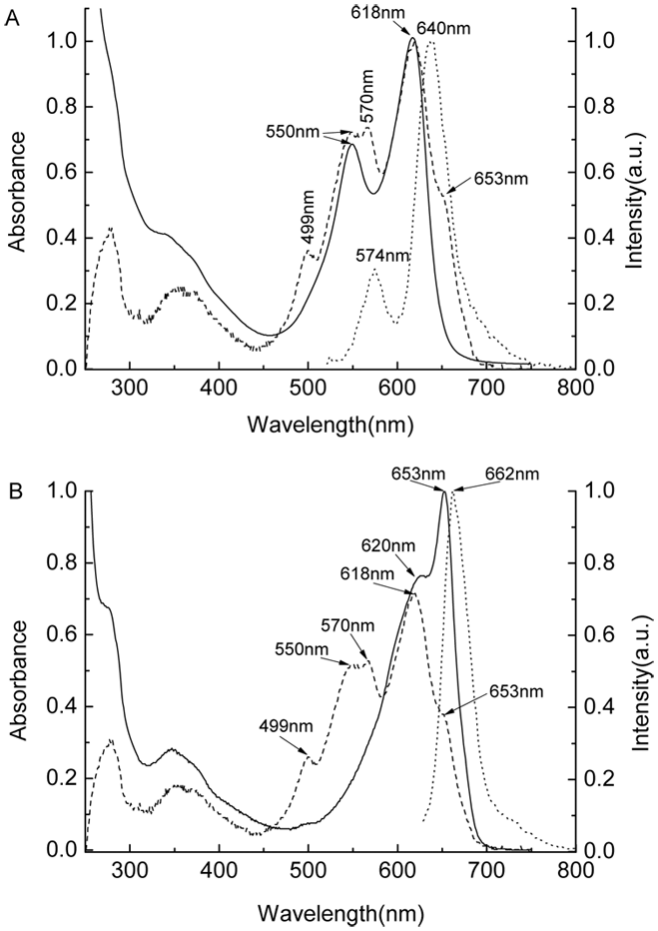
Absorption and fluorescence spectra of the R-PC and AP prepared by the PAGE in the HEPRES−Imidazole/Bis-tris and Bis-tris−HEPES−MES buffer systems. The absorption (solid line) and fluorescence (dot line) spectra of the prepared R-PCs and APs showed respectively in (A) and (B); the absorption spectrum of the sample, which was obtained by the ion exchange chromatography and used for the PAGE experiments, showed as dash lines both in (A) and (B). The absorption spectra were recorded in pH 7.0 phosphate buffers, and the fluorescence spectra of the R-PC and AP recorded in pH 7.0 phosphate buffer when excited respectively at 500 nm and 615 nm. The specific absorption and fluorescence bands of the R-PC (550 nm and 618 nm, and 574 nm and 640 nm), AP (653 nm and 620 nm, and 662 nm) and R-PE (499 nm and 570 nm) were labeled on the spectra.

Fig. 6 showed absorption and fluorescence spectra of the R-PC and AP components obtained by the native PAGE performed in the HEPRES−Imidazole/Bis-tris and Bis-tris−HEPES−MES buffer systems from the AP fraction (Fig. 6 dash line) prepared by the ion exchange chromatography. In the spectrum of the AP fraction (Fig. 6 dash line), the absorption bands at 499 nm and 570 nm were mainly come from R-PE components, the absorption at 550 nm and 618 nm were the specific bands of R-PC, and the absorption shoulder at 653 nm was attributed to AP components. The spectra of the prepared R-PC (Fig. 6A solid and dot lines) and AP (Fig. 6B solid and dot lines) showed the individual specific properties of natural complexes of R-PC (absorption maxima at 550 nm and 618 nm, and fluorescence emission at 640 nm) and AP (absorption maximum at 653 nm and fluorescence emission at 662 nm). This demonstrated that the buffer systems established for the native PAGE performed in pH 7.0 and 6.5 were successful in the preparation of phycobiliproteins in minor content, especially those sensitive to alkaline environments.

**Figure 7:**
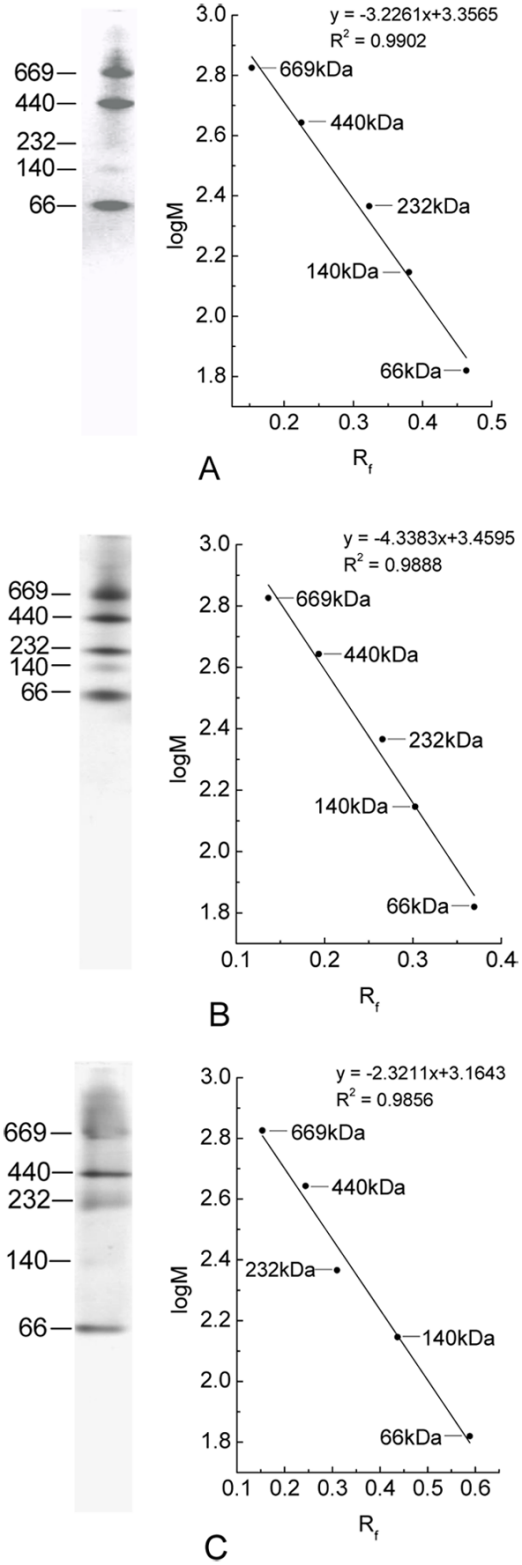
The resolution of high molecular mass protein markers (native) by the PAGE in the three buffer systems. (A) Bis-tris−HEPES−MES buffer systems; (B) HEPES−Imidazole/Bis-tris buffer systems; (C) Imidazole−Acetic acid buffer systems. The PAGE was performed with a gradient resolving gel of 5%–20% in pH 7.0 and a stacking gel of 3% in pH 6.0. The band patterns of each PAGE (left) were visualized by Coomassie Blue G-250.

**Figure 8:**
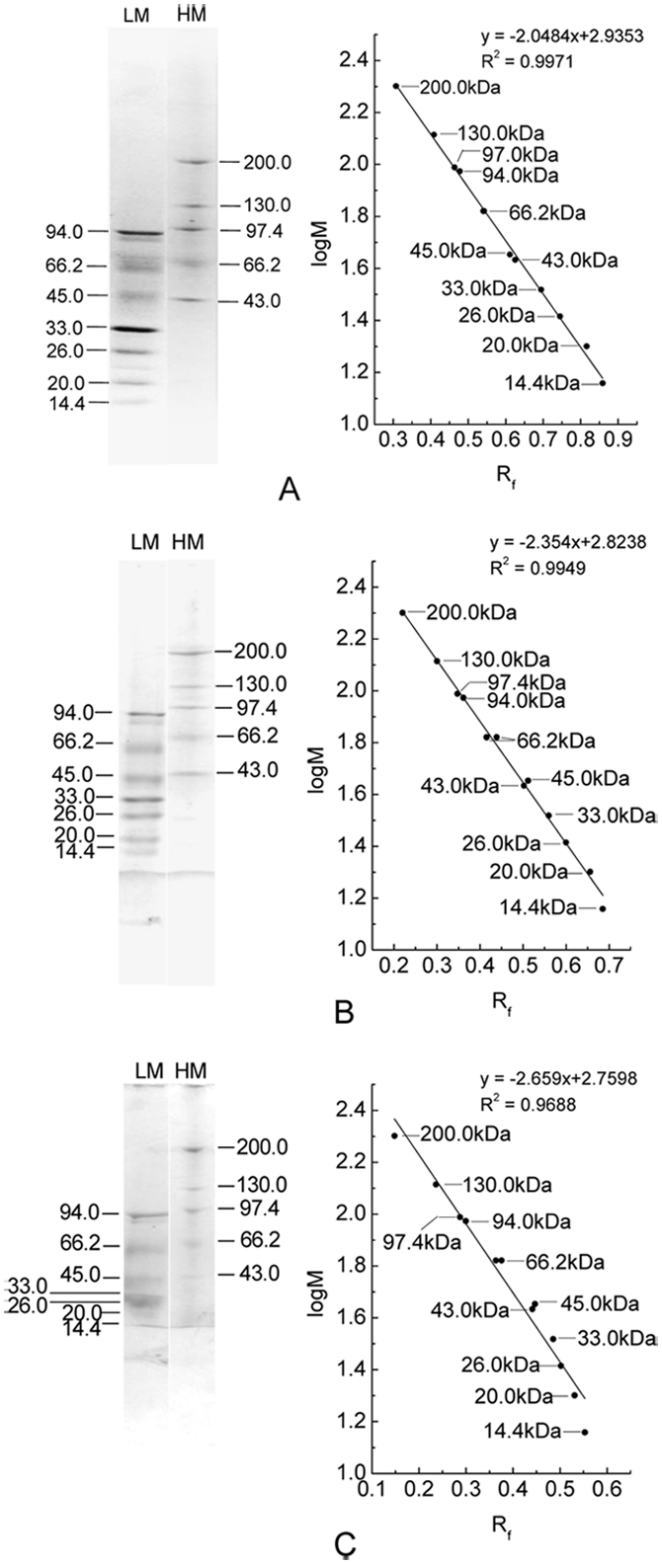
The resolution of high and low molecular mass protein markers (SDS) by the PAGE in the three buffer systems. (A) Bis-tris−HEPES−MES buffer systems; (B) HEPES−Imidazole/Bis-tris buffer systems; (C) Imidazole−Acetic acid buffer systems. The PAGE was performed with a gradient resolving gel of 5%–20% in pH 7.0 and a stacking gel of 3% in pH 6.0, and 0.1% SDS was added in cathode buffers. The band patterns of each PAGE were visualized by Coomassie Blue G-250.

In addition, separation of high molecular mass protein markers used in native PAGE were performed by the established PAGE modes with the three buffer systems of Bis-tris−HEPES−MES, HEPES−Imidazole/Bis-tris, Imidazole−Acetic acid in pH 7.0. As shown in Fig. 7, the five marker proteins were satisfactorily separated and the linear relationship between their logM and R_f_ was clearly exhibited by the calibration curves of logM versus R_f_. Moreover on the basis of the results showed by Fig. 8, protein markers of high and low molecular masses employed in SDS-PAGE were also adequately resolved by the three PAGE modes when 0.1% SDS was added in cathode buffers. Each of the PAGE modes gave acceptable calibration curves of logM vs. R_f_ (Fig. 8). These results demonstrate that the established buffer systems can have wider application in protein separation by PAGE.

## Discussion

During the Tris−Gly PAGE performing, the pH in its resolving gel and stacking gel is 9.5 and 8.3 [30], respectively; it means that in most of time during the PAGE performed in Tris−Gly buffer system, proteins had to be subject to an alkaline environment of pH 9.5. The value of pH 9.5 is near the upper limit for the R-PE to tolerate the effect of alkaline conditions on the spectral properties [12], and it is one and a half pH units above the pH 8.0 upper limit of the R-PC from *P. urceolata* (S. 1 ans S. 2). Such an alkaline environment of pH 9.5, which the R-PC and AP had to suffer in the Tris−Gly PAGE, was the key factor to resulting in their color fading and fluorescence decreasing; some of the protein bands occurring on the lane of Coomassie Blue G-250 staining (Fig. 1A lane d) should virtually originate from the dissociation or denaturing of the R-PC and AP complexes throughout the Tris−Gly PAGE. Likewise, the pH 8.0 condition, which the R-PC and AP complexes were subjected to during the Tris−Barbital PAGE [30], accounted for their partial color fading and fluorescence decreasing. In contrast with the former two, the PAGE modes performed with the established buffer systems (Fig. 1C and 1D), the pH of which was measured no more than 7.5 during the electrophoresis, efficiently avoided the effect of alkaline conditions on the structure or configuration of phycobiliproteins.

The Imidazole−Acetic acid buffer systems exhibited good sample stacking and band separating effects in the native PAGE of phycobiliproteins (Fig. 1) by adjusting the combination of the stacking and resolving buffers in different pH with the resolving gels in different concentration, but they were proved not to be suitable for phycobiliproteins owing to causing obvious dissociation of phycobiliproteins in the PAGE process. The dissociation may originate mainly from the effect of imidazole in higher concentration on phycobiliprotein complexes, for it can be efficiently eliminated by substituting the gel buffers of Imidazole−Acetic acid for those of HEPES−Imidazole (Fig. 2) where the imidazole concentration decreases ∼3/12 times in pH 7.0/6.5 resolving gels and ∼26/110 times in pH 6.0/5.4 stacking gels.

For the HEPES−Imidazole buffer systems, the loaded sample containing R-PC, AP and R-PE proteins showed the three resolved bands in the stacking gels of the native PAGE, and the three bands were piled up from the first to the last in a sequence of red R-PE, sky blue AP and blue R-PC. In the resolving gels of the PAGE, however, the protein bands became gradually broader with their running, giving an acceptable but undesirable result. The band broadening is assumed to relate to the different migrating behavior of HEPES ions with respect to the ions of imidazole and acetic acid as well as the inadequate sample stacking.

Compared with the two buffer systems mentioned above, the Bis-trsi−Acetic acid buffer systems showed the most efficient concentrating of samples in stacking gels. This demonstrated that the sample-concentrating effect originated from the state of isotachophoresis between the leading and tailing ions was sufficiently established in the stacking gels [29], [30]. Although the loaded sample stacked as a thin thread was acceptably resolved and separated in resolving gels, the PAGE running could not maintain the state in which the current or voltage of the running PAGE was continuously adjustable much longer than ∼90 minutes. This limitation is assumed to come from the running behavior of Bis-tris ions with respect to that of acetic acid ones, for it was not observed in the PAGE performed in the imidazole and HEPES systems.

Based on the PAGE where the Bis-tris−Acetic acid buffers were combined with the resolving gel buffers of the HEPES−Imidazole buffer system, the HEPES−Bis-tris and the Bis-tris−HEPES−MES buffer systems were designed and both of them used the common cathode and anode solutions of Tricine−Bis-tris and Bis-tris−Acetic acid buffers. By regulating the proportions of HEPES, MES and acetic acid to Bis-tris in the resolving and stacking gel buffers of different pH according to the experiment results, the analysis and preparation of the AP and R-PC components from the AP and R-PC fractions obtained by the column chromatography were optimally achieved in cooperation with the concentration modification of resolving gels. This achievement should be attributed to the migrating behavior of buffer component ions which is compatible with that of sample proteins in a certain electric field that is exerted on the PAGE gels in definite pH buffers.

Besides the differences of proteins in net static charge decided on the pH values of PAGE buffer systems, the differences of proteins with various molecule mass, size or shape in migration resistance decided on the pore sizes of resolving gels with different concentration is another crucial factor to determine the effectiveness of PAGE separation. In the experiments, when the resolving gel was selected to be 7%, the three proteins of R-PE, R-PC and AP moved in the whole time of the PAGE in pH 7.0 (Fig. 4A) and 6.5 (Fig. 1D and Fig. 3) in order from first to last: the R-PE, the AP and the R-PC. When the PAGE performed with the concentration of resolving gels higher than 9%, as showed in Fig. 4B and S. 3B, the three proteins ran in the same order as they in the 7% gel PAGE at the beginning of the proteins moving in the resolving gel; but with the electrophoresis continuing, the AP band gradually went through and over the R-PE band. These facts demonstrate that the R-PE, R-PC and AP migrate in the 7% resolving gel mainly on the basis of their differences in their net charge, which depend on their differences in pI, and the resistance of the gel pores, which is closely related to molecular mass, size and shape, is not large enough to affect the established migration order. In contrast, the resistance exerted on the hexamer R-PE (240 kDa) by the pores of the resolving gel with concentration ≥ 9% increases to such a degree that the R-PE migration speed is adequately decreased; consequently, the trimer AP (∼130 kDa) band the migration of which is less affected by the pore resistance can move over the R-PE band and is clearly resolved in front of the R-PE. For the PAGE without a stacking gel (Fig. 5), the AP band moved in front as soon as it ran into the 9% resolving gel because it was no need for the AP to take time to overcome the migration order of the three proteins which was originally established based on their net charge in the stacking gel. Thus this PAGE gives more convenience to the preparation of AP trimers.

In conclusion, the establishment of the discontinuous native PAGE performed in pH 7.0 and 6.5, which was favorable to the analysis and preparation of AP and R-PC components sensitive to alkaline environments, was achieved based on the experimental results of the three designed buffer systems. Furthermore, the buffer systems employed for the discontinuous native PAGE in this research, including the Imidazole−Acetic acid one, may provide useful references for some other proteins that are sensitive to alkaline environments. In addition, considering that the net charge of proteins is easier to exhibit larger differences in pH environments relatively closer to their pIs, the discontinuous native PAGE established in this work may provide another selection more favorable to resolving acidic proteins with approximately pIs ≤6.0, especially to those not to be effectively separated by the native PAGE performed normally in weak alkaline buffer systems. Therefore, these native PAGE modes carried out with the three buffer systems in pH 7.0 and 6.5 are promising complements to the native PAGE modes performed in the alkaline pH buffers of Tris−Gly and Tris−Barbital systems.

## Supporting Information

Figure S1 Variations of R-PC absorption spectrum in different pH solution. The absorption spectra were recorded in various 50 mM buffer solutions of equal R-PC concentration different in pH from 3.0 to 10.6 at room temperature after the R-PC was added in the buffer solution of a certain pH for 30 min. The solutions in pH 3.0, 4.0, 5.0 and 6.0 were prepared with citric acid and sodium citrate, the solutions in pH 7.0 and 8.0 were prepared with sodium dihydrogen phosphate and disodium hydrogen phosphate and the solution in pH 9.0, 10.0 and 10.6 were prepared with glycine and NaOH.
(DOC)

Figure S2 Variations of R-PC fluorescence emission spectrum in different pH solution. The fluorescence emission spectra were recorded in various 50 mM buffer solutions of equal R-PC concentration different in pH from 3.0 to 10.6 at room temperature after the R-PC was added in the buffer solution of a certain pH for 30 min. The solutions in pH 3.0, 4.0, 5.0 and 6.0 were prepared with citric acid and sodium citrate, the solutions in pH 7.0 and 8.0 were prepared with sodium dihydrogen phosphate and disodium hydrogen phosphate and the solution in pH 9.0, 10.0 and 10.6 were prepared with glycine and NaOH.
(DOC)

Figure S3 The native PAGE of the R-PC and AP fraction performed in Imidazole-Acetic acid buffers. The PAGE with the same stacking gel of 3% in pH 5.4 was performed in the Imidazole−Acetic acid buffers when the resolving gels of 6.5% in pH 6.5 (A) and 8%–16% in pH 7.0 (B) were employed. (a) showed the bands of native state in grey scale; (b) and (d) showed the fluorescent bands under UV-light at 365 nm; (c) and (e) showed the bands after Coomassie Blue G-250 staining. The sample was prepared by the gel filtration.
(DOC)
