# Supplementary material for: Separation of Native Allophycocyanin and R-Phycocyanin from Marine Red Macroalga Polysiphonia urceolata by the Polyacrylamide Gel Electrophoresis Performed in Novel Buffer Systems
Source: PLoS One. 2014 Aug 28;9(8):e106369. doi: 10.1371/journal.pone.0106369 (PMC4148431; doi:10.1371/journal.pone.0106369)
Supplement: Figure S1 — Variations of R-PC absorption spectrum in different pH solution. The absorption spectra were recorded in various 50 mM buffer solutions of equal R-PC concentration different in pH from 3.0 to 10.6 at room temperature after the R-PC was added in the buffer solution of a certain pH for 30 min. The solutions in pH 3.0, 4.0, 5.0 and 6.0 were prepared with citric acid and sodium citrate, the solutions in pH 7.0 and 8.0 were prepared with sodium dihydrogen phosphate and disodium hydrogen phosphate and the solution in pH 9.0, 10.0 and 10.6 were prepared with glycine and NaOH. (DOC) [file pone.0106369.s001.doc]

S.1 gave the absorption spectrum variations of the R-PC trimer prepared from red macroalga *P. urceolata*. The R-PC complex, as shown in S.1, exhibited almost equal absorbance at 550 nm and 617 nm in pH from 8.0 to 6.0, and as the solution pH ≤ 4.0 especially > 8.0 the absorbance at 550 nm and 617 nm obviously decreased with the pH value changing. The values of the R-PC specific absorption decreasing with pH changing from 8.0 to 10.6 were fairly greater than those from 6.0 to 4.0.

S. 1 **Variations of R-PC absorption spectrum in different pH solution.** The absorption spectra were recorded in various 50 mM buffer solutions of equal R-PC concentration different in pH from 3.0 to 10.6 at room temperature after the R-PC was added in the buffer solution of a certain pH for 30 min. The solutions in pH 3.0, 4.0, 5.0 and 6.0 were prepared with citric acid and sodium citrate, the solutions in pH 7.0 and 8.0 were prepared with sodium dihydrogen phosphate and disodium hydrogen phosphate and the solution in pH 9.0, 10.0 and 10.6 were prepared with glycine and NaOH.
