# Supplementary material for: Separation of Native Allophycocyanin and R-Phycocyanin from Marine Red Macroalga Polysiphonia urceolata by the Polyacrylamide Gel Electrophoresis Performed in Novel Buffer Systems
Source: PLoS One. 2014 Aug 28;9(8):e106369. doi: 10.1371/journal.pone.0106369 (PMC4148431; doi:10.1371/journal.pone.0106369)
Supplement: Figure S2 — Variations of R-PC fluorescence emission spectrum in different pH solution. The fluorescence emission spectra were recorded in various 50 mM buffer solutions of equal R-PC concentration different in pH from 3.0 to 10.6 at room temperature after the R-PC was added in the buffer solution of a certain pH for 30 min. The solutions in pH 3.0, 4.0, 5.0 and 6.0 were prepared with citric acid and sodium citrate, the solutions in pH 7.0 and 8.0 were prepared with sodium dihydrogen phosphate and disodium hydrogen phosphate and the solution in pH 9.0, 10.0 and 10.6 were prepared with glycine and NaOH. (DOC) [file pone.0106369.s002.doc]

Likewise, as shown in S. 2, the R-PC specific fluorescent emission at 639 nm was seemly no change within pH 7.0 to 8.0. But the intensity of the 639 nm emission showed obviously decreasing as the R-PC solution pH decreased from 7.0 to 3.0 and increased from 8.0 to 10.6, and the decrease scope of the fluorescence intensity was much greater from pH 8.0 to 9.0 than from pH 7.0 to 5.0.

From the results showed in S. 1 and S. 2, it can be concluded that the R-PC trimeric complex from red macroalga *P. urceolata* has some configuration parts which relate closely to the optimal configuration of chromophores and they are more sensitive to alkaline environments than acidic ones.

S. 2 **Variations of R-PC fluorescence emission spectrum in different pH solution.** The fluorescence emission spectra were recorded in various 50 mM buffer solutions of equal R-PC concentration different in pH from 3.0 to 10.6 at room temperature after the R-PC was added in the buffer solution of a certain pH for 30 min. The solutions in pH 3.0, 4.0, 5.0 and 6.0 were prepared with citric acid and sodium citrate, the solutions in pH 7.0 and 8.0 were prepared with sodium dihydrogen phosphate and disodium hydrogen phosphate and the solution in pH 9.0, 10.0 and 10.6 were prepared with glycine and NaOH.
