# Supplementary material for: Separation of Native Allophycocyanin and R-Phycocyanin from Marine Red Macroalga Polysiphonia urceolata by the Polyacrylamide Gel Electrophoresis Performed in Novel Buffer Systems
Source: PLoS One. 2014 Aug 28;9(8):e106369. doi: 10.1371/journal.pone.0106369 (PMC4148431; doi:10.1371/journal.pone.0106369)
Supplement: Figure S3 — The native PAGE of the R-PC and AP fraction performed in Imidazole-Acetic acid buffers. The PAGE with the same stacking gel of 3% in pH 5.4 was performed in the Imidazole−Acetic acid buffers when the resolving gels of 6.5% in pH 6.5 (A) and 8%–16% in pH 7.0 (B) were employed. (a) showed the bands of native state in grey scale; (b) and (d) showed the fluorescent bands under UV-light at 365 nm; (c) and (e) showed the bands after Coomassie Blue G-250 staining. The sample was prepared by the gel filtration. (DOC) [file pone.0106369.s003.doc]

Fig. S 3 showed the component analysis of the R-PC fraction from the filtration by the native PAGE performed with imidazole−acetic acid buffers. The PAGE with a resolving gel of 6.5% in pH 6.5 (Fig. 1 A) exhibited three main bands: trimeric R-PC, hexameric R-PE and a band located in front of others, whereas after the loaded R-PC sample was concentrated several times, a AP band occurred just before the R-PE and some more bands were resolved in the front of the gradient gel (Fig. 1 B).

S. 3. **The native PAGE of the R-PC and AP fraction performed in Imidazole-Acetic acid buffers.** The PAGE with the same stacking gel of 3% in pH 5.4 was performed in the Imidazole−Acetic acid buffers when the resolving gels of 6.5% in pH 6.5 (A) and 8%-16% in pH 7.0 (B) were employed. (a) showed the bands of native state in grey scale; (b) and (d) showed the fluorescent bands under UV-light at 365 nm; (c) and (e) showed the bands after Coomassie Blue G-250 staining. The sample was prepared by the gel filtration.
